# Supplementary material for: Identification of photoperiod-induced specific miRNAs in the adrenal glands of Sunite sheep (Ovis aries)
Source: Front Vet Sci. 2022 Jul 22;9:888207. doi: 10.3389/fvets.2022.888207 (PMC9354845; doi:10.3389/fvets.2022.888207)
Supplement: Supplementary file 1 [file Data_Sheet_1.ZIP › Supplementary file/miRDeep2 file.zip!/miRDeep2 file/Know_Structure/SPLP42A2/oar-mir-410.pdf]

miRBase precursor : oar-mir-410
Total read count : 998
oar-miR-410-5p read count : 8
oar-miR-410-3p read count : 987
remaining reads : 3

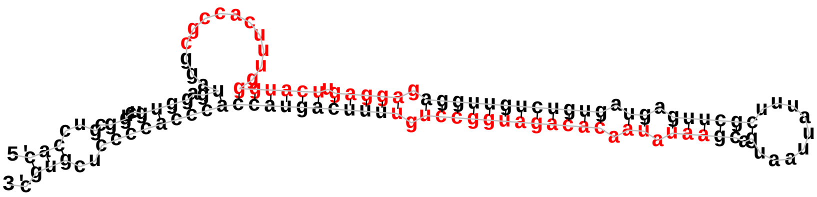

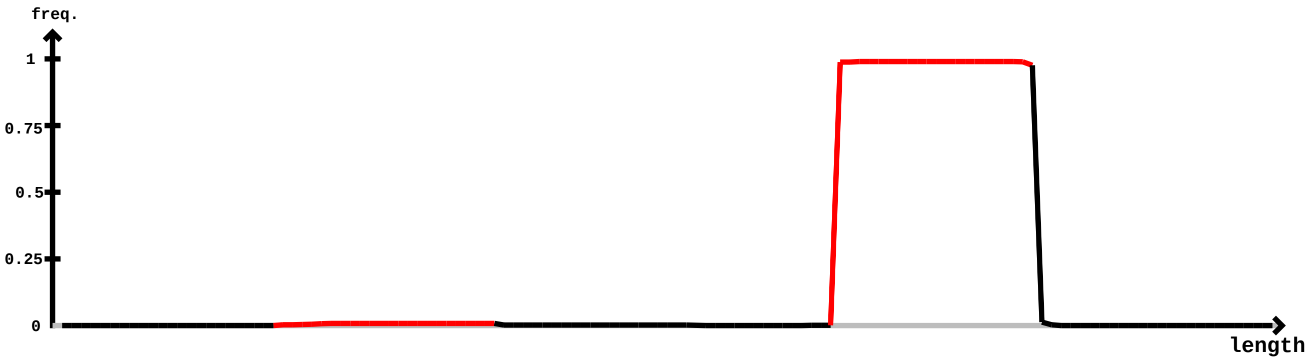

oar-miR-410-5p

oar-miR-410-3p

```
5'- caccugcggucuguggaguaggcgccacuuuggguacuugaggagagguugucugugaugaguucgcuuuauuaaugacgaauauaacacagauggccuguuuucaguaccacccaccccucgugc   -3'    exp
    (((..(.(((..((((........))))....((((((.(((((.((((((((((((.((.((((((.........).))))).)).)))))))))))).)))))))))))....))).)..))).   reads      mm
    ......................cgccacuuuggguacuugaggag.................................................................................   3          0
    ........................ccacuuuggguacuugaggag.................................................................................   1          0
    .........................cacuuuggguacuugaggag.................................................................................   1          0
    ..........................acuuuggguacuugaggag.................................................................................   2          0
    ...........................cuuuggguacuugaggag.................................................................................   1          0
    .............................................agguugucugugaugaguuc.............................................................   1          0
    .............................................agguugucugugaugaguucg............................................................   1          0
    .............................................................................acgaauauaacacagauggccugu.........................   1          0
    ................................................................................aauauaacacagauggccu...........................   1          0
    ................................................................................aauauaacacagauggccug..........................   13         0
    ................................................................................aauauaacacagauggccugu.........................   960        0
    ................................................................................aauauaacacagauggccuguu........................   10         0
    ................................................................................aauauaacacagauggccuguuu.......................   1          0
    ..................................................................................uauaacacagauggccuguuu.......................   2          0
```
